# Efficacy of Sovodak in the Management of Patients Co-infected with HIV/HCV

**DOI:** 10.34172/apb.2020.080

**Published:** 2020-08-09

**Authors:** Mohammad Hossein Somi, Bita Sepehri, Zeinab Nikniaz, Roya Sedghi

**Affiliations:** Liver and gastrointestinal Diseases Research Center, Tabriz University of Medical Sciences, Tabriz, Iran.

**Keywords:** Daclatasvir, Hepatitis C, HIV, Sofosbuvir, Sovodak

## Abstract

***Purpose:*** Sofosbuvir (SOF) and daclatasvir (DOC) are suggested for the treatment of hepatitis C virus (HCV) in patients with concomitant HCV and human immunodeficiency virus (HIV). In 2016, Sovodak tablet a combination of SOF and DOC was introduced. In the present study we assessed the effectiveness of SOF in the treatment of HCV in patients co-infected with HIV.

***Methods:*** A total of 26 HCV patients co-infected with HIV received SOF for 3 months. One patient did not adhere to the drug protocol and was removed from the final analysis. The blood sample for qualitative polymerase chain reaction (PCR) was obtained after treatment and sustained virological response (SVR) was calculated.

***Results:*** Twenty five patients finished the study. The mean patients’ age was 44.16±6.21 years. About 72% of participants had HCV genotype 1a, 8% genotype 1b, and 20% genotype 3a. After 3 months of intervention with Sovodak, the SVR12 was about 96%. None of the patients reported any adverse events.

***Conclusion:*** For the first time, the results of the present study showed that Sovodak had high SVR12 in HCV patients co-infected with HIV. However, for a precise conclusion, there is a need for larger studies and an equal number of patients with different virus genotypes.

## Introduction

It has been estimated that more than 4 million people are co-infected with hepatitis C (HCV) and human immunodeficiency virus (HIV) worldwide.^1^ It is most prevalent among injecting drug users.^2^ Previous studies have indicated that more than 90% of HIV infected injecting drug users are co-infected with HCV.^3-5^ Moreover, according to the results of a systematic review in Iran, the prevalence of HIV/HCV co-infection among injecting drug users was about 10%.^6^

It has been shown that HIV/HCV co-infection increases the mortality rate compared with patients with mono-infection. HIV/HCV co-infection accelerates the progression of the disease to end-stage liver disease and liver failure and it is the major cause of morbidity and mortality in HIV-infected people.^7,8^ So, effective treatment of HCV in HIV/HCV co-infection is the priority to prevent HCV-related liver diseases. However, the treatment of these patients is complicated due to the interaction of antiretroviral drugs with direct-acting antivirals (DAAs).^9^

Daclatasvir (DAC) and sofosbuvir (SOF) are among the DAAs with the lowest potential for interactions with antiretrovirals.^10,11^ Some earlier studies investigated the effect of SOF/DAC in the treatment of HCV in patients with HIV/HCV co-infection and achieved high sustained virological response (SVR).^9,12^ Luetkemeyer et al studied the effect of 12-week treatment with SOF/DAC in HIV/HCV (genotype 1) co-infection and witnessed that SVR12 was about 97%.^9^ In another study in patients with HIV/HCV (genotype 1-4) co-infection, the SVR12 was 97% in naïve patients and 98.1% in previously treated patients.^12^

Recently, for the ease of use, DAC and SOF were combined as a single drug (Sovodak) in Iran and different studies evaluated the efficacy of this drug in HCV patients. Merat et al studied the influence of 3-month supplementation with Sovodak in the treatment of HCV patients (genotype 1 & 3) with cirrhosis and observed that SVR12 was 97.87%.^13^ In another study, Mehdipour et al assessed the influence of Sovodak on HCV genotype 1 treatment in naïve and previously treated patients and reported that SVR12 was 100%.^14^

Although two studies have assessed the effect of Sovodak in HCV patients, there is no current study to investigate its effect on patients co-infected with HIV/HCV. Hence, this study was designed to evaluate the efficacy of Sovodak in patients with HCV-HIV co-infection.

## Materials and Methods

### Patients

This is a before-after study. The HCV patients co-infected with HIV were selected from Behavioral Disorders Clinic of Tabriz University of Medical Sciences in 2017. The patients were included if they were male and were infected with HIV and HCV and also previously treated with antiretroviral therapies. The patients who had kidney disease (defined as eGFR <30 mL/min) and cirrhosis, used amiodarone, had previous treatment failure with other HCV treatment regimen, and those who had no interest to use Sovodak were excluded from the study.

Based on the inclusion criteria, 34 patients referring to Behavioral Disorders Clinic had the criteria to participate in the present study. Since their HCV was diagnosed by HCV-antibody testing, quantitative PCR was done for excluding the false-positive results. For this purpose, 5 cc of blood sample was obtained and quantitative viral load was done using COBAS Taqman HCV V2 (Roche Diagnostics, Indianapolis, IN) with a lower limit of quantification of 15 IU/mL.^15^ Seven patients were excluded due to false-positive results and one patient did not consent to participate in the study. Accordingly, 26 patients entered the study.

### Intervention

The Sovodak (Rojan Pharmaceuticals, Tehran, Iran) regimen was administered orally once a day for 12 weeks. Two types of Sovodak are available including SOF/DAC 400/60 and 400/90. The patients received different dose regimens of Sovodak based on their antiretroviral therapies. In this regard, 19 patients were treated with efavirenz or nevirapine as antiretroviral therapies. So, they received Sovodak containing SOF/DAC 400/90 and others received the dose of SOF/DAC 400/60.

### Measurements

Before the initiation of the intervention, the information regarding the age, duration of the disease, and medications were recorded. Moreover, laboratory analyses including white blood cell (WBC) and haematologic profiles were performed using an automatic cell counter. Alanine aminotransferase (ALT) and Aspartate aminotransferase (AST) were measured by enzymatic colorimetric assay. Eight patients had a low platelet level (<150 000). Thus, complementary investigations including measurement of international normalized ratio and albumin and liver sonography were done to ensure that the patients with cirrhosis were not included.

### Efficacy assessment and safety monitoring 

After 12 weeks of treatment, the real-time HCV assay was used to determine the HCV RNA level (Roche Molecular Systems). The minimum limits of quantification and detection of this method were 25 IU/mL and 20 IU/mL.

The treatment efficacy was defined as the absence of detectable HCV RNA in the serum 12 weeks after the end of the treatment (SVR12). A questionnaire about the side effects of drug including nausea, vomiting, skin rash, headache, fatigue, and other side effects was completed.

### Statistical Analysis

Data were analyzed using SPSS 19. The Kolmogorov-Simonov test was used for assessing the distribution of data. The continuous variables were reported as mean and standard deviation and qualitative data were presented as frequency and percentage. A binomial test was used to compare the SVR12 found in the present study with that of the previous study in HCV patients.

## Results and Discussion

**Figure 1:**
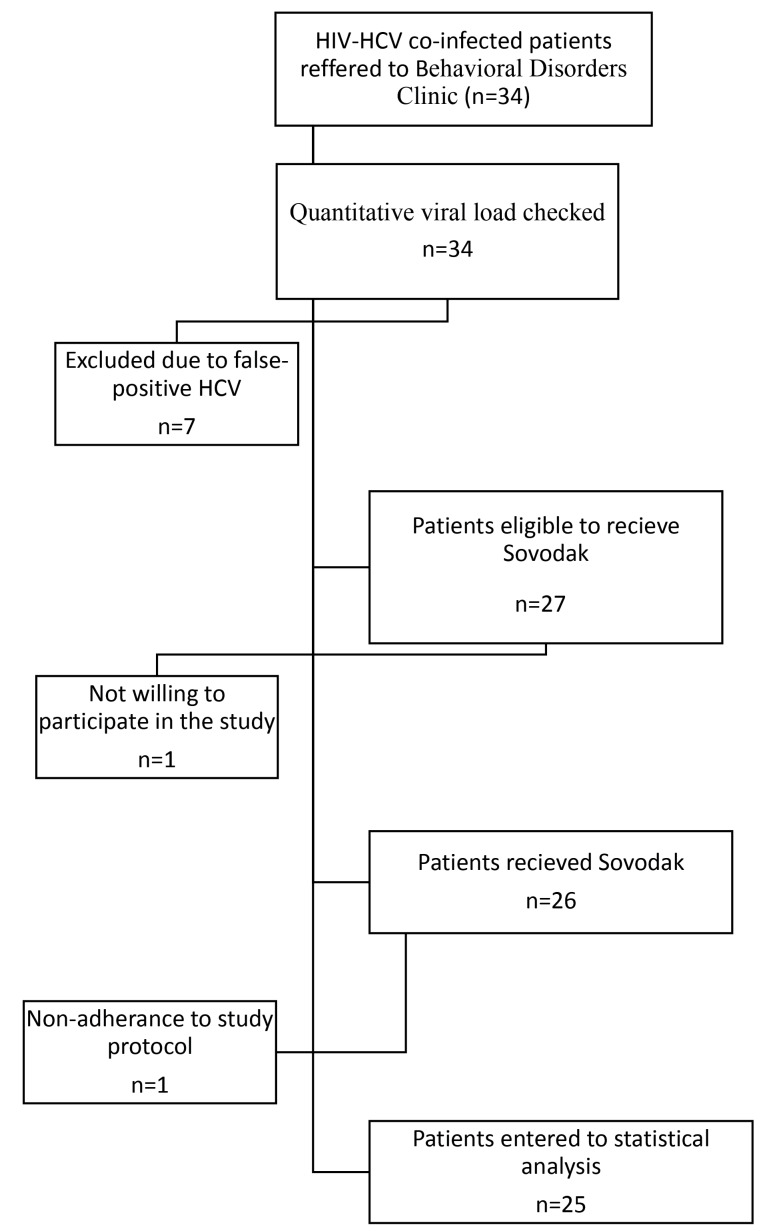
The flowchart of patient’s recruitment.

As Figure 1 shows, 26 patients were included in this study. One patient did not adhere to the treatment protocol and was excluded from the analysis. As shown in Table 1, the mean age of the participants was 44.16 ± 6.21 years. All patients were in the active phase of HIV infection. The mean duration of HIV infection was about 9.7 ± 3.1 years and the mean duration of ART (antiretroviral treatment) was about 4.3 ± 1.2 years. The mean CD4 level was 250 cell/cumm. The mean laboratory parameters were in the normal range. About 72% of participants had virus genotype of 1a, 8% genotype 1b, and 20% genotype 3a.

**Table 1:** The baseline characteristics of the patients (n = 25)

**Variable**	**Mean ± SD**
Age (y)	44.16 ± 6.21
WBC (per µL)	4732 ± 1272.70
Hemoglobin (g/dL)	14.95 ± 1.47
Platelet (per µL)	187880 ± 55902
AST (U/mL)	23.71 ± 11.83
ALT (U/mL)	25.04 ± 9.96
HCV viral load	4888076 ± 4501169
**HCV Genotype**	**No. (%)**
Genotype 1a	18 (72.0)
Genotype 1b	2 (8)
Genotype 3a	5 (20)

WBC: white blood cell; AST: aspartate amino transferase; ALT: alanine aminotransferase; HCV: hepatitis C virus; SD: standard deviation.

**Figure 2:**
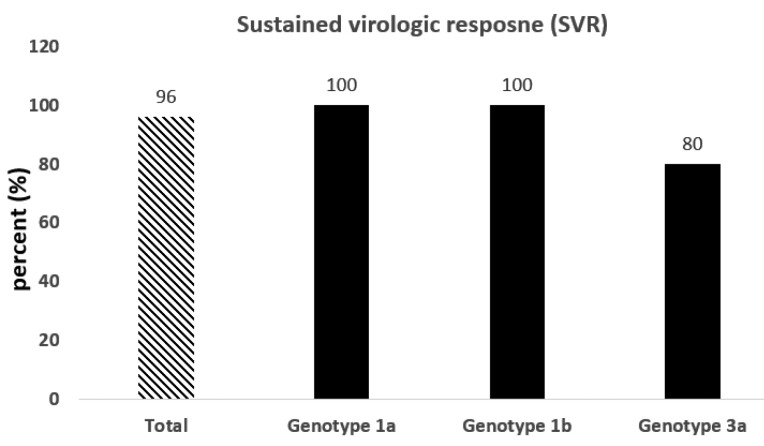
The sustained virologic response stratified by genotype (n = 25).

As presented in Figure 2, after three months of intervention with Sovodak, the SVR12 was about 96% (24 out of 25 patients). One of the patients who did not respond to Sovodak had HCV genotype 3a and received the Sovodak with a dose of 400/90. The efficacy of Sovodak has been shown in patients with HCV in a previous study (SVR: 97.87%).^13^ The results of the binomial test showed that there was no significant difference in SVR12 found in the present study and SVR12 reported in the HCV mono-infection patients (*P* = 0.18). Moreover, the high SVR (96%) in our study was in agreement with the results of some previous studies. For example, Luetkemeyer et al studied the effect of SOF/DAC in 150 patients co-infected with HIV/HCV (genotypes 1-4) (50 treatment-naïve and 100 treatment-experienced patients) and reported that SVR12 was 97%.^9^ In another study, Wyles et al revealed that in patients with genotypes 1-4, administration of 400 mg SOF and 60 mg DAC resulted in the SVR12 of 97% in the treatment-naïve and SVR12 of 98.1% in the treatment-experienced patients.^12^ The SVR in the present study is higher than the reported SVR in a previous study. In a real-life experience, Rockstroh et al reported the SVR of 92% in HIV/HCV patients with advanced liver diseases receiving DAC plus SOF.^16^ This difference could be related, in part, to the fact that the characteristics of included patients in these studies were different. In the present study, the patients with advanced liver diseases and hepatitis B infection were excluded. Besides, according to the results, Sovodak is effective as much as other DAAs in HIV/HCV co-infection. For example, Osinusi et al reported that SVR was 98% in 50 patients who received Ledipasvir and SOF for HCV treatment.^17^

According to the results of the present study, no side effect was reported for Sovodak. This finding was in accordance with the results of previous studies that did not report any side effects for combination therapy of SOF and DAC.^9,12^ In a previous study in HCV patients, it was observed that about thirty percent of patients treated by Sovodak had reported fatigue.^13^ The discrepancy between the results may be due to the differences in the characteristics of the included patients. Merat et al included HCV patients with cirrhosis; however, in the present study the patients with cirrhosis were excluded.

This study had some limitations. The overall sample size for the present study was low and the number of patients was very low in genotype 3 group. Moreover, we did not include females and patients with cirrhosis. Therefore, we cannot have a definite conclusion in genotype 3, females, and cirrhotic patients.

## Conclusion

In conclusion, for the first time, the results of the present study showed that SVR12 was 96% in HCV patients co-infected with HIV. Considering the limitation of the present study, there is a need for larger studies and an equal number of patients with different virus genotypes for confirming these preliminary results. For further analysis, it is also recommended to investigate the effectiveness of Sovodak in HCV and HIV patients who had cirrhosis.

## Ethical Issues

The ethical approval was obtained from the ethics committee of Tabriz University of Medical Sciences (IR.TBZMED.REC.1397.439) and an informed written consent was obtained from all participants before conducting the study.

## Conflict of Interest

Authors declare no conflict of interest in this study.

## Acknowledgments

The authors wish to thank the Liver and Gastrointestinal Diseases Research Center of Tabriz University of Medical Sciences for financial support. This study has been extracted from a fellowship thesis in Gastroenterology by Roya Sedghi (No. 60297).
